# Network Pharmacology and Experimental Validation of the Anti-Inflammatory Effect of Modified Qianzheng Powder in Atherosclerosis

**DOI:** 10.2174/0113862073376112250602092950

**Published:** 2025-07-03

**Authors:** Nianpei Yin, Hui Luo, Jie Feng, Weisheng Zhan, Zheng Zhou, Ying Yang

**Affiliations:** 1 Department of Clinical Medicine, North Sichuan Medical College, Nanchong 637000, Sichuan, China;; 2 Department of Cardiovascular Disease, Affiliated Hospital of North Sichuan Medical College, Nanchong 637000, Sichuan, China;; 3 Department of Thoracic Surgery, Second Clinical College of Nanchong North Sichuan Medical College, Nanchong Central Hospital, Nanchong 637000, Sichuan, China

**Keywords:** Network pharmacology, molecular docking, atherosclerosis, inflammatory, modified qianzheng powder, IL-6/STAT3 signaling pathway

## Abstract

**Objective:**

This study aims to validate the hypothesis that Modified Qianzheng Powder (MQZP) exerts a protective effect on atherosclerosis (AS) by targeting macrophage-associated inflammatory pathways through a multi-target approach.

**Materials and Methods:**

The active compounds and targets of MQZP were identified through TCMSP, HERB, and published literature. AS-related targets were extracted from disease databases. Using Venny 2.1.0, intersection targets were obtained, followed by PPI network construction and topological analysis to identify core therapeutic targets of MQZP for AS. Metascape facilitated GO and KEGG enrichment analyses. Molecular docking validated core target-compound interactions, with experimental verification of network pharmacology results.

**Results:**

We identified 124 active compounds and 417 potential therapeutic targets for atherosclerosis. Key bioactive constituents included cyclo(D)-Pro-(D)-Phe, aurantiamide, and beauverilide A, with TP53, SRC, STAT3, and AKT1 as core targets. GO and KEGG enrichment analyses revealed 3,417 biological processes and 238 signaling pathways. Molecular docking confirmed stable binding between core targets and compounds. *In vitro*, MQZP exhibited no significant cytotoxicity, effectively reducing ox-LDL-induced macrophage lipid accumulation and downregulating the levels of IL-1β, TNF-α, and MCP-1.

**Discussion:**

In summary, we found a variety of active ingredients of MOZP, which interfere with multiple targets of AS through multiple pathways. *In vitro* experiments verified that MOZP can reduce lipid accumulation in macrophages, reduce inflammation levels, and inhibit the IL-6/STAT3 signaling pathway, thereby exerting its anti-atherosclerosis effect.

**Conclusion:**

In this paper, the molecular mechanism of MOZP against AS has been preliminarily explored; nonetheless, the therapeutic mechanism needs to be further investigated.

## 1 INTRODUCTION

Cardiovascular diseases (CVD) include various conditions affecting the heart and circulatory system, such as coronary artery disease (CAD), hypertension, stroke, and heart failure, among others. These diseases are a major cause of global mortality and disease burden [1]. The Global Burden of Disease report indicates that the prevalence of cardiovascular metabolic diseases is steadily rising around the globe, with an upward trend in the number of CVD-associated deaths [2]. Atherosclerosis (AS), a chronic vascular condition, is primarily caused by the accumulation of plaques (comprising cholesterol, lipids, calcium, and other substances) within the arteries, leading to the thickening and narrowing of arterial walls [3, 4]. Plaque formation within blood vessels often causes vascular obstruction, triggering acute events such as myocardial ischemia/infarction or stroke [5]. Additionally, the incidence and progression of atherosclerosis (AS) are recognized as critical factors contributing to the onset of cardiovascular diseases (CVD). Diabetes, hypertension, dyslipidemia, obesity, and smoking are some of the major risk factors for AS [6]. Additionally, extensive research on AS pathogenesis has enhanced the general understanding of clinical phenomena such as lipid metabolism disorders, endothelial cell dysfunction, and inflammation [7, 8]. Although statins and antiplatelet medications can prevent and treat AS, they are associated with certain limitations in terms of efficacy and safety [9, 10]. Therefore, it is imperative to develop alternative treatments for AS.

Traditional Chinese medicine (TCM) has a long history and unique advantages in the treatment of atherosclerosis (AS) [11]. TCM emphasizes the principle of "syndrome differentiation and treatment", which involves balancing Yin and Yang, unblocking meridians, activating blood circulation, removing blood stasis, and expelling wind and dampness to treat AS. TCM treatments often involve the use of herbal medicine, acupuncture, and tuina [11-13]. Traditional Chinese herbal formulations such as the Tongxinluo capsule, Xuefu Zhuyu decoction, and Buyang Huanwu decoction have been used to clinically treat AS [14]. Jiang *et al*. discovered that the Tongxinluo capsule can delay AS progression by inhibiting endothelial cell apoptosis [15]. Furthermore, the Xuefu Zhuyu decoction exerts protective effects against AS through anti-inflammatory and antioxidant pathways, as well as lipid metabolism regulation [16]. Additionally, several other herbal medicines have been widely used as adjunctive therapies for AS. Paeoniae Radix Rubra helps slow the progression of atherosclerosis by dilating blood vessels, enhancing microcirculation, improving blood flow, and preventing lipid deposition and thrombus formation [17]. Salvia miltiorrhiza has strong antioxidant and anti-inflammatory effects, which help mitigate endothelial cell damage in arterial walls and prevent further progression of atherosclerosis [18, 19]. In the future, with the modernization of TCM research and the development of integrated Chinese and Western medicine treatments, the potential of TCM in the treatment of atherosclerosis is expected to be more widely applied and validated. For example, the combined use of statins with Salvia miltiorrhiza and Pueraria lobata can more effectively reduce blood lipid levels and alleviate side effects [20].

Qianzheng powder, originating from the "YANG's Jiacang Fang," can dispel wind, transform phlegm, promote meridian circulation, and alleviate spasms. The modified formula known as Modified Qianzheng Powder (MQZP), adapted from the original formula based on individual patient conditions, comprises Baifuzi (Typhonii Rhizoma), Baijiangcan (Bombyx Batryticatus), Quanxie (Scorpio), Chishao (Paeoniae Radix Rubra), Danggui (Angelicae Sinensis Radix), Chuanxiong (Chuanxiong Rhizoma), and Jixueteng (Spatholobi Caulis). Clinically, MQZP is primarily used to treat facial neuritis and vascular diseases. According to research, Qianzheng powder components could exert anti-inflammatory effects, ameliorate ischemia and hypoxia-associated circulation disorders, and enhance immune function. Specifically, Baifuzi could reduce inflammation, counteract oxidative stress, and enhance endothelial cell function [21], and mitigate ischemic stroke by acting on calcium channels [22]. On the other hand, Baijiangcan and its active components could exert pharmacological effects, including antimicrobial, antiviral, anti-inflammatory, antioxidant, hypolipidemic, hypoglycemic, and anticoagulant properties [23, 24]. Furthermore, besides its potent anti-tumor and antithrombotic effects [25, 26]. Quanxie may also suppress inflammatory macrophage activity and prevent endothelial cell damage [27, 28].

Interleukin-6 (IL-6), an inflammatory cytokine, is involved in AS progression. It could upregulate adhesion molecules in endothelial cells, stimulate platelet activation, and reduce plaque stability [29]. Furthermore, inflammatory response activation *via* the IL-6/STAT3 signaling pathway could further exacerbate AS progression [30].

This study employs a multi-target approach to validate the hypothesis that MQZP exerts protective effects on AS by targeting inflammatory pathways in macrophages. The aim is to identify the bioactive components of MQZP, evaluate its therapeutic potential, and elucidate its molecular mechanisms in AS. Herein, we collected the effective components and therapeutic targets of MQZP through network pharmacological analysis. We also employed molecular docking techniques and performed *in* *vitro* experiments to validate the mechanisms behind the protective action of MQZP against AS.

## 2 MATERIALS AND METHODS

### 2.1 Gathering the Active Components and Targets of MQZP

First, we retrieved the active components of each medicinal herb contained in MQZP from the TCMSP Database (https://tcmsp.91medicine.cn/#/home). At the same time, we selected compounds with OB ≥ 30% and DL ≥ 0.18 [31]. Subsequently, herbs absent from the TCMSP database were searched for in the HERB database (http://herb.ac.cn/). We also obtained additional information for specific animal-derived herbs such as "Quanxie" and "Baijiangcan". The higher components of SwissADME were selected for subsequent analysis. Finally, published literature was consulted to supplement information on the formula's effective components. The obtained chemical constituents were subsequently submitted to the SwissTargetPrediction database (http://swisstargetprediction.ch/) to identify drug targets, excluding those with a confidence score of 0.

### 2.2 Collection of AS targets

Using "Atherosclerosis" as the keyword, four databases [GeneCards (https://www.genecards.org/), DisGeNET (https://www.disgenet.org/), OMIM (https://omim.org/), and DrugBank (https://go.drugbank.com/)] were searched for AS-related targets [32]. Only protein targets associated with AS in "Homo sapiens" were selected [33]. All duplicate entries were merged to obtain a consolidated list of AS-related targets.

### 2.3 Identifying Common Targets, Building the "Herb-Ingredient-Target" Network, and Screening for Key Effective Components

The pertinent targets of MQZP and AS were uploaded to an online graphing platform to create a Venn diagram, illustrating the overlapping targets, which were regarded as the primary potential targets of MQZP for treating AS. Using Cytoscape 3.9.1 software, the " herb-ingredient-target " network was constructed to explore the associations between the herbal medicine, its active ingredients, and potential targets. The topological parameters of each node in the network were obtained using the Analyze plugin in Cytoscape software [34]. The top 10 compounds with the highest degree values were selected, which we consider to be the key components likely responsible for MQZP's therapeutic effects on AS.

### 2.4 Construction of the protein-protein interaction (PPI) network and screening of key targets

The intersection targets were input into the STRING database (https://cn.string-db.org/), with the species set to "Homo sapiens," the minimum interaction score set to the highest confidence (0.90), and isolated protein nodes hidden, while the remaining settings were left at default [35]. The resulting data were then exported in TSV format and imported into Cytoscape 3.9.1 for PPI network visualization. First, network topology analysis was performed using the CytoNCA plugin, with parameters including betweenness centrality (BC), closeness centrality (CC), and degree centrality (DC). The selection criteria were the median values of the three parameters, followed by two rounds of screening to obtain a core PPI network. The top 10 targets with the highest degree values were selected as key candidate targets for further analysis. Next, the CytoHubba plugin was used to identify the top 10 genes with the highest scores from the PPI network using the MNC algorithm. The intersection of the top 10 targets from both algorithms was taken, and the overlapping targets were considered the key targets for MQZP in the treatment of AS. Finally, the MCODE tool within Cytoscape was employed to carry out modular analysis of the PPI network. The PPI network was then segmented into multiple clusters based on MCODE scores, with the most significant module in the network identified as the highest-scoring cluster.

### 2.5 Gene Ontology (GO) and Kyoto Encyclopedia of Genes and Genomes (KEGG) Enrichment Analyses

We used the Metascape database (http://metascape.org/) to perform GO and KEGG enrichment analyses, identifying potential therapeutic targets of MQZP for AS [36]. The selection included the top ten GO terms and twenty KEGG pathways with the lowest p-values, which were then uploaded to an online graphing platform for visualization. A network diagram depicting the primary pathways and targets associated with AS treatment using MQZP was created with Cytoscape 3.9.1 software.

### 2.6 Molecular Docking Validation

Molecular docking was performed on the proteins TP53, SRC, STAT3, AKT1, JUN, and IL-6, along with their corresponding compounds. The 3D structures of the proteins were downloaded from the PDB database (http://www1.rcsb.org/), and the protein structures were inspected using PyMOL 2.3.0 software in preparation for docking. The ligand small molecule structures were obtained from PubChem (https://pubchem.ncbi.nlm.nih.gov/), and the small molecule structures were optimized using the MMFF94 force field in OpenBabel 3.1.1 software to obtain the lowest energy conformations. Hydrogenation of the proteins and small molecules, as well as flexibility treatments, were performed using AutoDock Tools 1.5.6 Docking parameters for each protein were set using the Grid tool. The docking method was set to semi-flexible docking with an exhaustiveness value of 25, and the docking algorithm used was the Lamarckian genetic algorithm. Molecular docking was carried out using AutoDock Vina 1.2.0 software, and the binding free energies and docking result files were obtained [37].

### 2.7 Preparation and Extraction of Active Ingredients from MQZP

Prepare the TCM materials included in MQZP (comprising Baifuzi, Baijiangcan, Quanxie, Chishao, Danggui, Chuanxiong, and Jixueteng). The specific preparation method was as follows: First, the aforementioned Chinese herbs were soaked in cold ultrapure water ten times their weight for 1 h. Subsequently, the mixture was brought to a boil in high heat and simmered with a low flame for 40 minutes before collecting the resulting medicinal liquid. Next, an additional eight-fold volume of water was incorporated, and the mixture was simmered for 30 minutes. The final step involved collecting the resulting medicinal liquid. Subsequently, the two batches of medicinal liquids were combined, filtered through gauze, and simmered until reaching a 1 g/ml concentration. The resulting medicinal liquid was frozen at -80 °C overnight and subsequently freeze-dried using a vacuum freeze dryer, yielding 2.58 g of freeze-dried powder.

### 2.8 Cell Culture

RAW264.7 (Cell Line Type: Mouse Monocytic Macrophage Cell Line Catalog Number: CL-0190 Source: Procell) cells were maintained in DMEM medium enriched with 10% FBS and 1% penicillin-streptomycin. The cells were incubated in a moisture-controlled environment at 37°C with 5% CO2. Afterwards, the MQZP powder was reconstituted in serum-free DMEM and diluted to the required concentration. The experimental groups consisted of the control group, ox-LDL group, and MQZP group, with three replicates per group. All experimental conditions, including the composition of the culture medium and the experimental environment, were strictly controlled, ensuring that the conditions for all three groups were identical, except for the intervention factors.

### 2.9 Cell Viability Assay

We utilized the Cell Counting Kit-8 (CCK-8, Biosharp, China) to assess how ox-LDL affects the viability of RAW264.7 cells and to investigate the impact of MQZP on cell survival. To start, RAW 264.7 cells were initially seeded at a density of 6×10^3^ cells per well in a 96-well plate during their logarithmic growth phase. After the cells adhered, the corresponding drugs were added and left to act for 24 hours. Following this, 10 μL of the CCK-8 working solution was added to each well. Absorbance was measured at 450 nm with a microplate reader after a 2-hour incubation period.

### 2.10 Oil Red O Staining

Cultivate the cells following the previously established cell seeding method. After cell adherence, drugs at corresponding concentration gradients were added per the experimental protocol. After a 24-hour exposure to the drug, the cells were rinsed twice with PBS, treated with the oil red O fixative solution (Solarbio, China) for 20 minutes, and then rinsed twice with distilled water. Subsequently, the cells were rinsed with 60% isopropanol for 20-30 seconds and treated with oil red O dye (Solarbio, China) for 15 minutes. After another 20-30 seconds of rinsing with 60% isopropanol until the interstitium became clear, the cells underwent five washes with water to eliminate any excess staining solution. Finally, an Oil Red O buffer was added for 1 min before covering the cells with distilled water. The intracellular lipid droplets were observed using a microscope, under which images were captured.

### 2.11 ELISA

The cells were treated with MQZP at 150 μg/mL for 1 hour, after which they were exposed to 200 μg/mL of ox-LDL for 24 hours. The cell culture supernatant was harvested for ELISA analysis to detect the concentration of IL-1β, TNF-α, and MCP-1 using their corresponding commercial ELISA assay kits (ZCIBIO Technology Co., Ltd, China).

### 2.12 RT-qPCR

Following the manufacturers' protocols, we isolated total RNA from RAW264.7 cells using an RNA extraction kit from Yeasen Biotechnology Co., Ltd. (China) and synthesized cDNA with a reverse transcription kit from TaKaRa Biotechnology Co., Ltd. (China). All primers were designed and synthesized by Shanghai Sangon Biotech Co., Ltd. (China), with their sequences provided in Table **1**. The mRNA expression levels of TP53, SRC, STAT3, and IL-6 in all groups were measured through RT-qPCR reactions using the TB Green Premix Ex Taq II kit (TaKaRa Biotechnology Co., Ltd., China). The relative mRNA expression levels were calculated using the 2-ΔΔCT method, with β-actin serving as the reference gene.

### 2.13 Western Blotting

IL-6 (Bioss, bs-0782R), p-STAT3 (abcam, ab76315), STAT3 (proteintech, 10253-2-AP), and β-actin (abclonal, AC026). To begin, RAW264.7 cells underwent treatment with RIPA lysis buffer (Biosharp, China) supplemented with 0.1% PMSF (Biosharp, China) for the extraction of total cellular proteins. The concentration of these proteins was determined using a BCA protein quantification kit from Beyotime (China). Protein samples were prepared by mixing them with 5× loading buffer (Biosharp, China) and heating at 95°C for 15 minutes to denature. The separated proteins were analyzed using SDS-PAGE (Biosharp, China) and transferred onto a PVDF membrane (Sigma-Aldrich, Germany). For blocking, the membrane was treated with 5% skimmed milk (Servicebio, China) and incubated overnight at 4°C with primary antibodies. After a washing step, secondary antibodies were added and incubated at room temperature for 2 hours. The resulting protein bands were subsequently captured and analyzed with Image-J software.

### 2.14 Statistical Analysis

Statistical analyses were conducted using SPSS version 27.0 and GraphPad Prism version 8.0. All experiments were conducted in triplicate. Unless stated differently, data were presented as mean ± standard deviation (SD). One-way ANOVA was used to evaluate differences among groups. For post hoc analysis, Dunnett’s T3 test was applied in cases of unequal variances, while Tukey’s test was used for pairwise multiple comparisons when variances were equal. Statistical significance was defined by a *p*-value lower than 0.05.

## 3 RESULTS

### 3.1 Screening of Relevant Targets of MQZP and AS

Herein, the TCMSP database was screened for 51 active compounds of MQZP with proven efficacy, including Baifuzi (3 types), Chishao (18 types), Chuanxiong (7 types), Danggui (2 types), and Jixueteng (21 types). Additionally, 18 active compounds from MQZP were successfully identified as effective through the HERB database, including Quanxie (2 types) and Baijiangcan (16 types). Furthermore, 56 key MQZP components were retrieved from existing literature, including Baifuzi (9 types), Baijiangcan (2 types), Quanxie (3 types), Chishao (7 types), Danggui (23 types), Chuanxiong (10 types), and Jixueteng (1 type). Details of MQZF are presented in Table **2**. Therefore, 124 effective ingredients were obtained, and some elements were present in two or more compounds (Additional file 1: Table **S1** details the included compounds). After excluding targets with credibility scores of 0 and merging duplicates, 1061 potential therapeutic targets for MQZP were discovered.

**Fig. (1):**
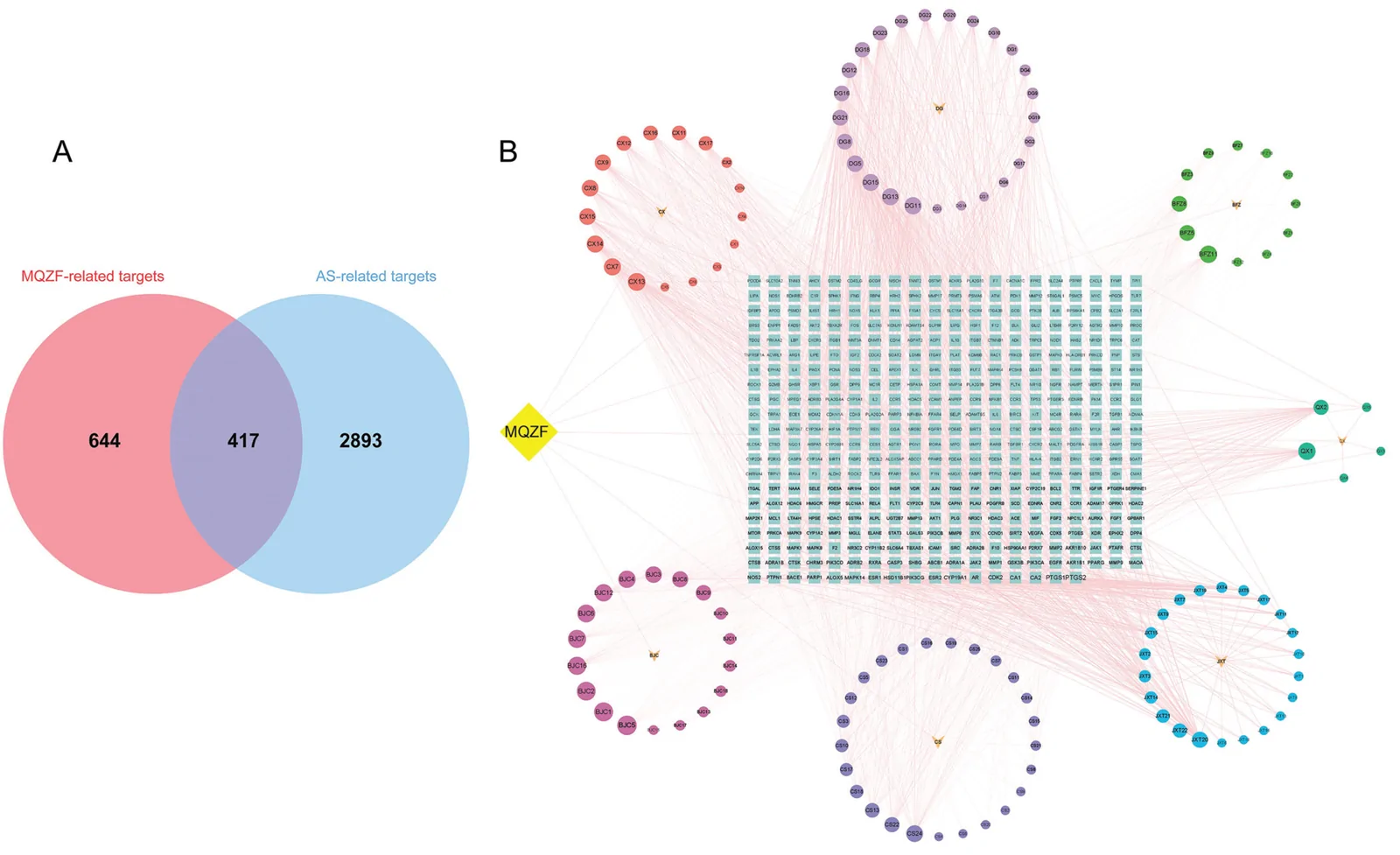
Venn diagram and “herb-ingredient-target” network diagram. (**A**) A Venn diagram illustrating the intersecting targets between MQZP and AS. (**B**) The “herb-ingredient-target” network diagram. The yellow rhombus indicates the MQZP compound formula, orange arrows represent 7 traditional Chinese medicines, individual circles within 7 circles represent 124 effective components, cyan squares represent 417 potential targets, and pink lines indicate interactions between various nodes.

Potential targets for AS were sourced from the 4 databases. Following the removal of duplicates, 3310 potential AS targets were identified. The Venn diagram highlighted 417 shared targets (Fig. **1A**).

### 3.2 The "Herb-Ingredient-Target" Network Analysis

The network, comprising 7 Chinese herbs, 124 active compounds, and 417 potential therapeutic targets, was constructed with Cytoscape 3.9.1 software (Fig. **1B**). This network comprised 549 nodes (one compound, seven Chinese medicines, 124 effective ingredients, and 417 potential therapeutic targets) and 2691 edges (representing interactions among various nodes). The number of edges connected to a node is indicated by its degree value. The cyclo(D)-Pro-(D)-Phe node had the highest degree value, linking 60 target nodes. Aurantiamide (degree=57), beauverilide a (degree=56), and ecdysone (degree=52) were among the ingredients with higher degree values. These components may be the key effective ingredients in AS treatment (Table **3** shows the topological parameters of the key effective ingredients).

### 3.3 PPI Network Analysis

**Fig. (2):**
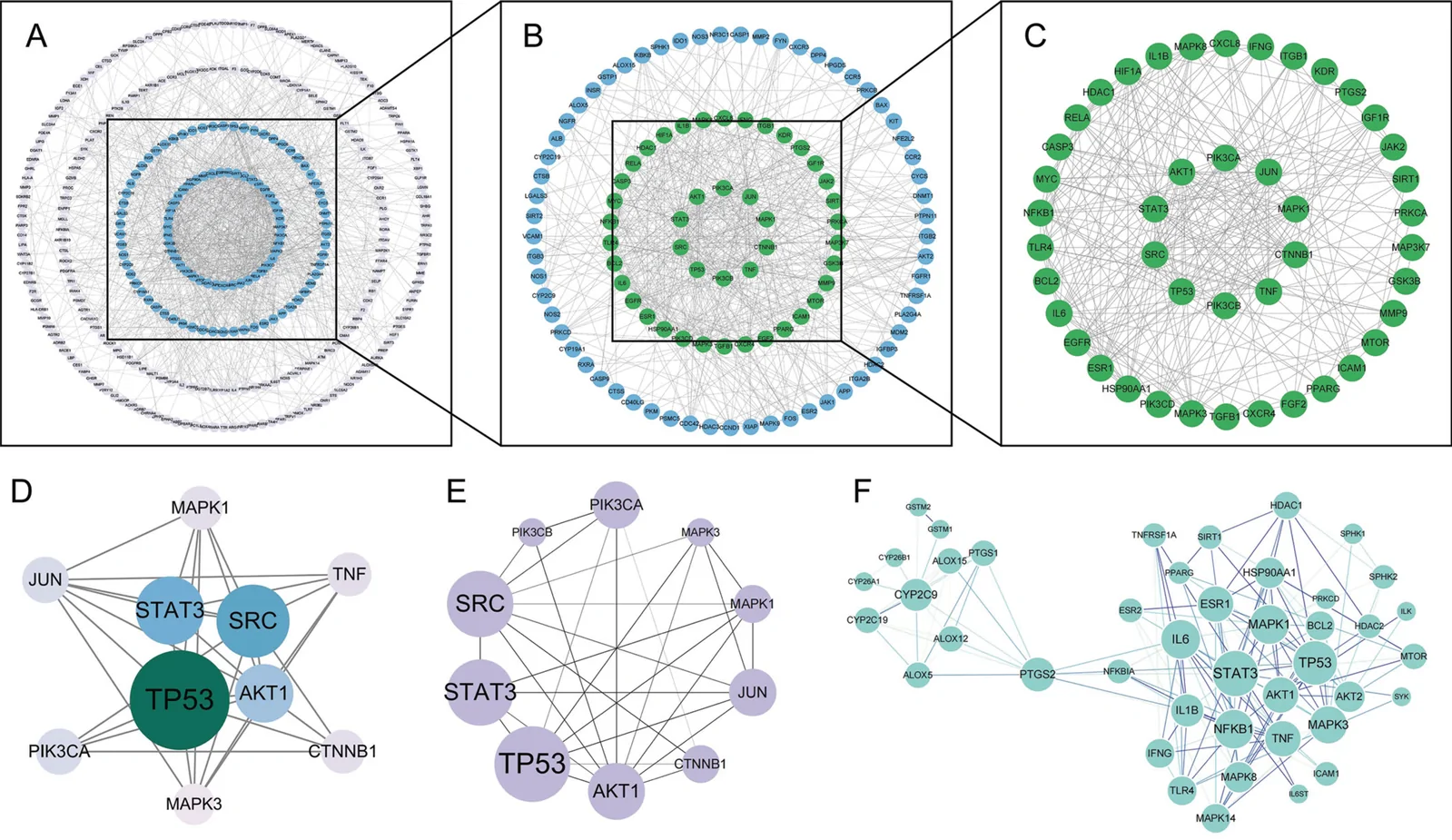
PPI Network Graph. (**A**) First round of screening. (**B**) Second round of screening. (**C**) Core PPI network graph. (**D**) First set of candidate key targets (top 10degree values in the core PPI network). (**E**) Second set of candidate key targets (top 10 based on CytoHubba plugin using the MNC algorithm). (**F**) MCODE Cluster 1.

Comprising 349 nodes and 1251 edges, the PPI network depicted protein names and their interactions. The initial visualization and analysis with Cytoscape 3.9.1 software led to the construction of the original PPI network. Using the CytoCNA plugin, a concentrated analysis and assessment of the PPI network was performed. Two rounds of screening were then performed based on the criteria established by respective medians. The first round retained targets meeting the criteria and labeled them in blue (Fig. **2A**; Criteria for selection: BC >164.16, CC >0.264, DC >5). Fig. (**2B**) depicts the targets (marked green) selected in the second screening (Criteria for selection: BC >1030.85, CC >0.30, DC >9). The network created after the second evaluation was considered the main PPI network (comprising 44 nodes and 269 edges), in which larger nodes corresponded to elevated degree values (Fig. **2C**). The top 10 targets are considered to be pre-selected key core targets (Fig. **2D**). Using the MNC algorithm, we identified the top ten-ranked nodes from the PPI network in the CytoHubba plugin, including TP53(51), SRC(41), STAT3(41), AKT1(36), PIK3CA(32), JUN(32), CTNNB1(31), MAPK1(31), PIK3CB(30), MAPK3(30) (Fig. **2E**). After intersecting the two sets of key alternative targets, nine final core targets were obtained (Additional file 1: Table **S2** Information of 9 core targets), including TP53, SRC, STAT3, AKT1, JUN, PIK3CA, MAPK1, CTNNB1, and MAPK3. These targets may be crucial in anti-AS MQZP effects. The MCODE plugin was employed to conduct module analysis of the PPI network, which revealed 17 distinct modules. Cluster 1 (Fig. **2F**, comprising 43 genes and 159 edges) attained the highest MCODE score of 7.571, implying that it represented the most significant within the PPI network.

### 3.4 GO and KEGG Pathway Enrichment Analyses

**Fig. (3):**
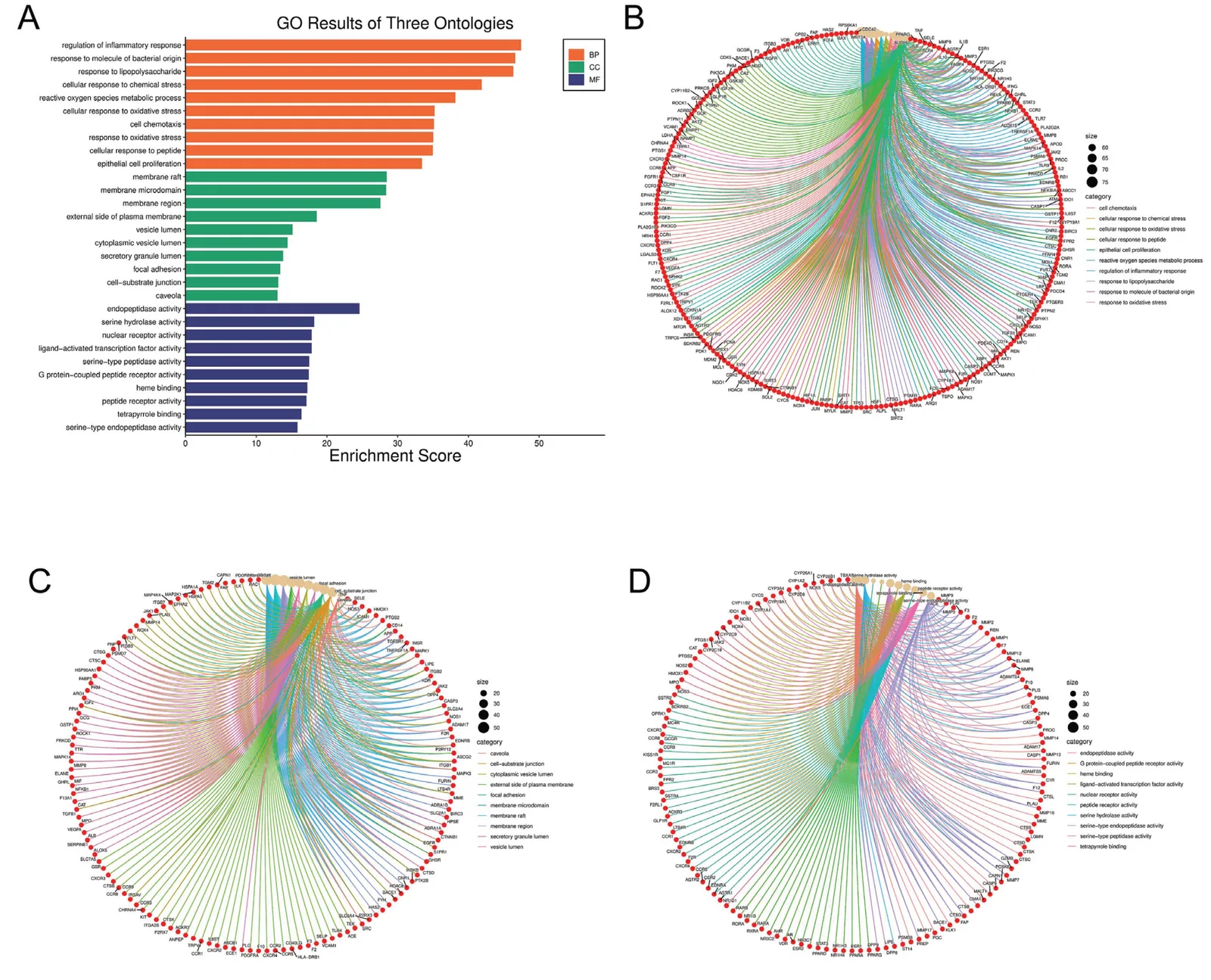
GO enrichment analysis of intersection targets. (**A**) Three bar charts displaying the outcomes of GO enrichment analysis. (**B**) The cnet diagram for BP. (**C**) The cnet diagram for CC. (**D**) cnet diagram for MF.

Herein, a total of 417 potential targets underwent GO and KEGG pathway enrichment analyses. The GO analysis outcomes encompassed three aspects: biological processes (BP), cellular components (CC), and molecular functions (MF). Based on the *p* < 0.05 and q < 0.05 criteria, 3417 BP, 107 CC, and 292 MF terms were obtained. The ten most significant terms for BP, CC, and MF were chosen based on p-values and visualized as bar graphs and cnet diagrams (Fig. **3**). Within the BP category, the intersecting targets were mainly linked to inflammatory response regulation, response to bacterial origin molecules, lipopolysaccharide response, cellular response to chemical stress, and reactive oxygen species metabolic processes. On the other hand, the CC were predominantly distributed in the membrane raft, membrane microdomain, membrane region, external side of the plasma membrane, and vesicle lumen. The results for MF indicated that the potential therapeutic targets were connected to various processes such as endopeptidase, serine hydrolase, nuclear receptor, ligand-activated transcription factor, and serine-type peptidase activities.

**Fig. (4):**
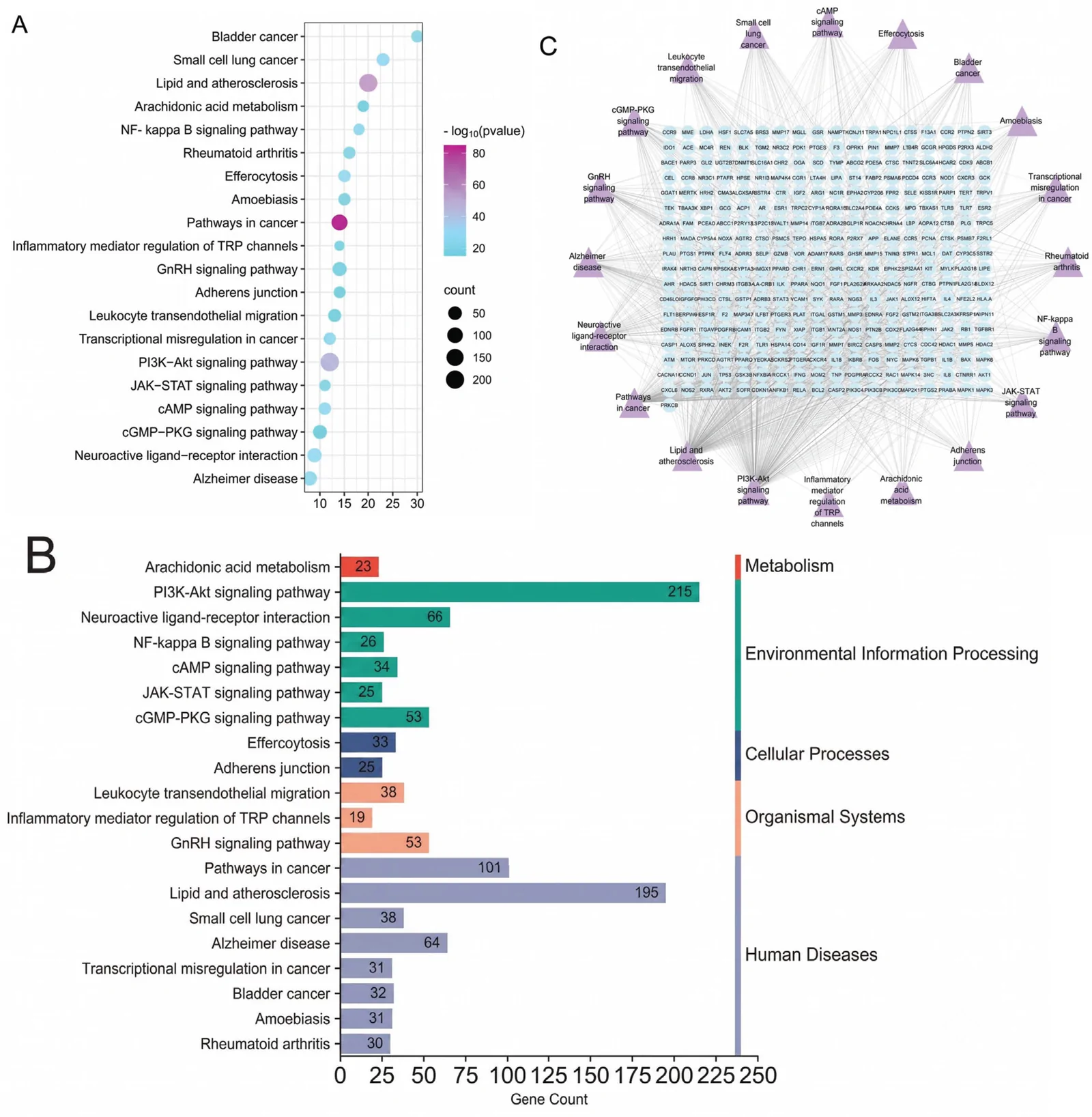
KEGG enrichment analysis of intersection targets. (**A**) Bubble chart illustrating the outcomes of top 20 KEGG enrichment analysis. (**B**) KEGG pathway secondary classification map. (**C**) The network diagram of the top 20 pathways and their corresponding targets.

In KEGG pathway analysis, 238 pathways were enriched (criteria: *p* < 0.05 and q < 0.05), from which the top 20 pathways were identified for visualization in a bubble plot (Fig. **4A**). In the plot, each point's size corresponds to the quantity of genes linked to that pathway, and the color signifies the degree of enrichment, transitioning from purple to blue as p-values decrease. At the same time, we classify the top 20 pathway maps (Fig. **4B**). Red represents metabolism, green represents environmental information processing, purple represents cellular processes, orange represents organismal systems, and gray represents human diseases. Cytoscape 3.9.1 software was subsequently employed to map the top twenty KEGG pathways with the lowest *p*-values and their corresponding targets, constructing a "key pathway-target" network for MQZP in AS treatment (Fig. **4C**). The KEGG enrichment analysis indicated a primary focus of the identified therapeutic targets on pathways associated with human diseases, including lipid and atherosclerosis, small cell lung cancer, and rheumatoid arthritis. Furthermore, signaling pathways, such as PI3K-Akt, NF-κB, cAMP, JAK-STAT, and cGMP-PKG, were significantly enriched. Cellular processes pathways, including efferocytosis and adherens junctions, were also enriched. Notably, targets are strongly associated with PI3K-Akt and lipid and atherosclerosis pathways. In AS treatment, MQZP demonstrated a protective effect characterized by diversification, multi-target intervention, multiple action pathways, and comprehensive regulation.

### 3.5 Analysis of Molecular Docking Results

Typically, a binding energy lower than 0 kcal/mol indicates that the receptor and ligand can spontaneously interact without the need for external energy, while a binding energy below -5 kcal/mol implies a robust binding affinity. This study revealed that TP53, SRC, STAT3, AKT1, JUN, and IL-6, as well as their corresponding compounds, showed favorable docking effects (Additional file 1: Table **S3**). The PyMOL 2.3.0 software was employed to visualize the docking results for TP53-Luteolin, SRC-ergotamine, STAT3-cyclo-D--Pro-D-Phe, and IL-6-Luteolin interactions, and Fig. (**5**) shows the detailed docking interactions.

### 3.6 MQZP Promotes RAW264.7 Cell Viability and Ameliorates ox-LDL-induced Lipid Accumulation

**Fig. (6):**
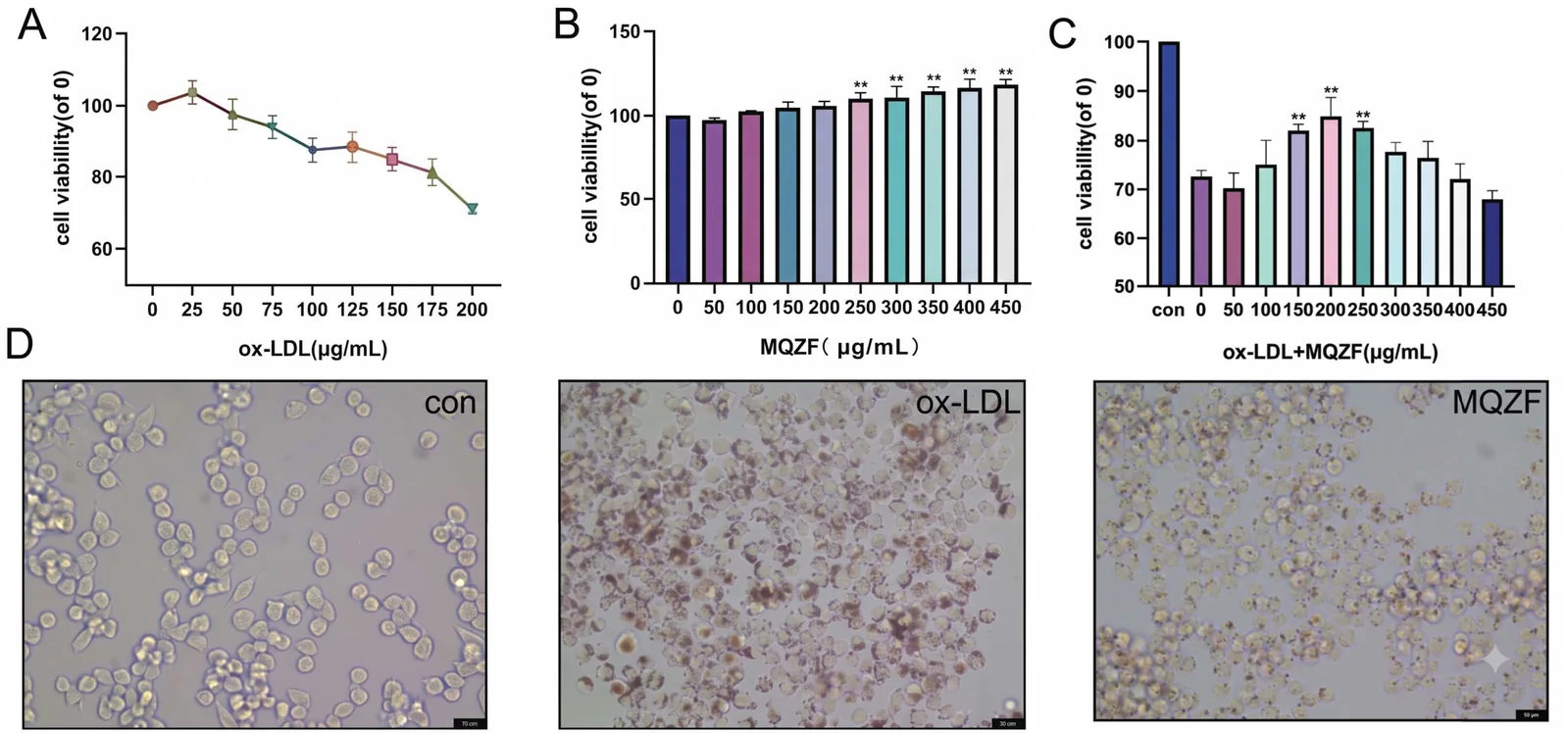
MQZP enhances the viability of RAW264.7 cells subjected to ox-LDL and reduces ox-LDL-induced lipid accumulation. (**A**) Viability of RAW264.7 cells treated with ox-LDL. (**B**) Viability of RAW264.7 cells treated with MQZP. (**C**) Viability of RAW264.7 cells co-treated with ox-LDL and MQZP. (**D**) Oil Red O staining. Data are expressed as mean ± SD, *P<0.05, **P<0.01 compared to the control group; #*P*<0.05, ##*P*<0.01 compared to the ox-LDL group.

In the early stages of atherosclerotic plaque development, the macrophage-induced LDL-C and ox-LDL phagocytosis, leading to foam cell formation, is pivotal in AS pathogenesis. Therefore, we developed an AS foam cell model by utilizing macrophages induced by ox-LDL. Specifically, RAW264.7 cells were cultured using various ox-LDL concentrations. The CCK8 assay results indicated that ox-LDL decreased cell viability, with viability falling below 0.8 at 200μg/mL (Fig. **6A**). Consequently, we selected the 200μg/mL ox-LDL concentration as the standard for subsequent experiments. We also investigated the impact of MQZP on cell survival. In comparison to the control group, cell survival in the MQZP groups (at all concentrations) remained 100% (except for 50 μmoL/L), implying that the examined MQZP doses exhibited no cytotoxic effects (Fig. **6B**). We treated ox-LDL-treated RAW264.7 cells with different doses of MQZP to investigate the specific therapeutic dose at which MQZP exerts its protective effects on RAW264.7 cells [38]. The results showed that MQZP, within the concentration range of 150 to 350 μg/L, was able to enhance cell viability (Fig. **6C**). Therefore, we selected 150 μg/L as the therapeutic dose for subsequent experiments.

To assess the effect of MQZP on lipid accumulation in RAW264.7 cells, Oil Red O staining was used. In comparison to the control group, the ox-LDL group showed a marked increase in lipid accumulation, confirming that the 200 μg/mL concentration of ox-LDL effectively induced an AS foam cell model. Furthermore, introducing 150 μmoL/L MQZP to the ox-LDL-induced foam cell model significantly decreased intracellular lipid accumulation (Fig. **6D**). Overall, our experimental results demonstrated that MQZP could enhance RAW264.7 cell viability and significantly reduce ox-LDL-induced lipid accumulation.

### 3.7 MQZP Reduced Inflammation by Inhibiting the IL-6/STAT3 Signaling Pathway in RAW264.7 Cells

**Fig. (7):**
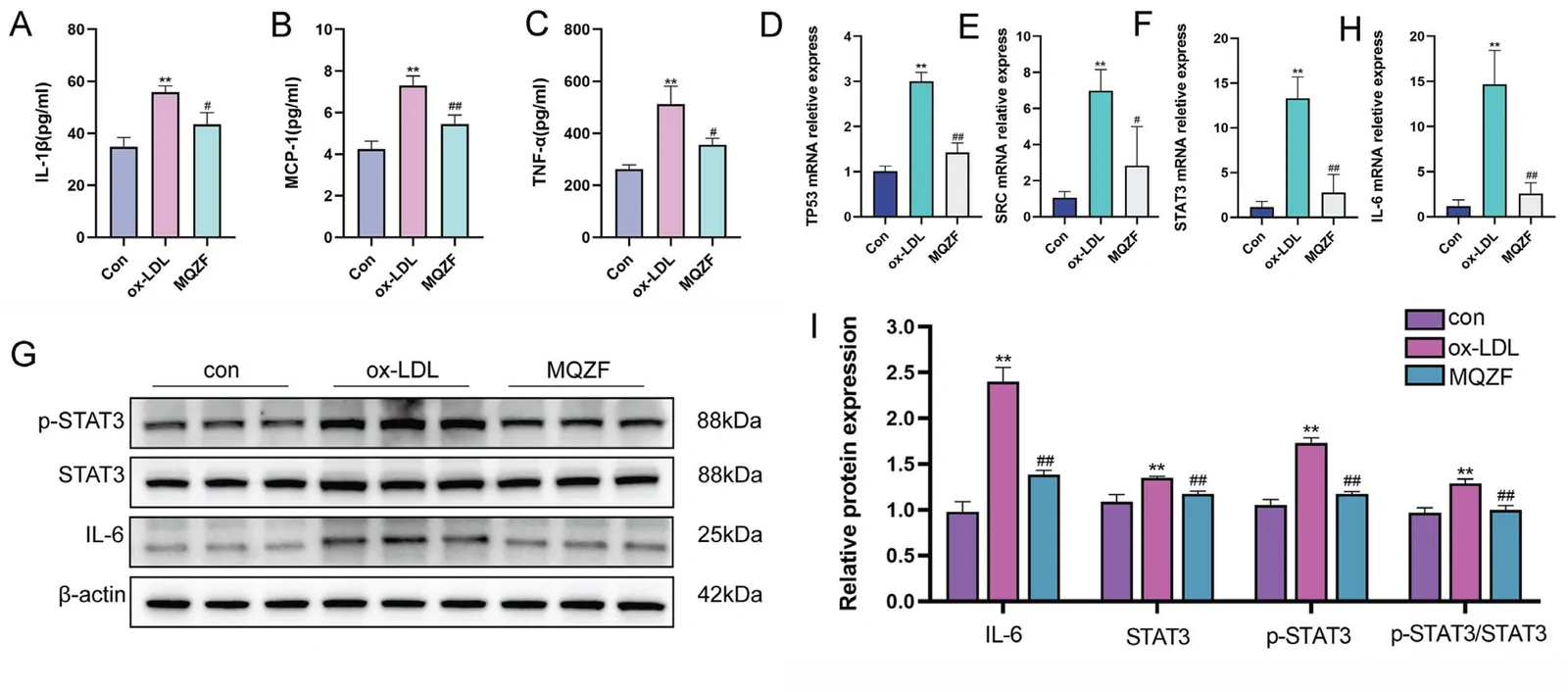
Effects of MQZP on ox-LDL-induced inflammatory cytokine secretion in RAW264.7 cells and its impact on predicted targets and pathways in AS. (**A-C**) The effect of MQZP on IL-1β, TNF-α, and MCP-1 secretion levels. (**D-G**) RT-qPCR assessment of TP53, SRC, STAT3, and IL-6 gene mRNA expression levels. (**H**) Western blot displaying the expression levels of IL-6, STAT3, and p-STAT3 proteins in cells derived from RAW264.7 (**I**) Quantitative analysis of protein blots using β-actin as the internal reference. Comparison of the ox-LDL group to the control group and the MQZP group to the ox-LDL group. Data are presented as mean ± SD, *P<0.05, **P<0.01 *vs.* control group; #*P*<0.05, ##*P*<0.01 *vs.* ox-LDL group.

The development of foam macrophages is recognized as a trigger for an inflammatory response that accelerates the progression of AS. Furthermore, inflammatory mediators like IL-1β, IL-6, MCP-1, and TNF-α are identified as key factors contributing to AS. To investigate the anti-inflammatory properties of the MQZP TCM formulation, we used ELISA kits to measure the concentrations of various inflammatory markers within the cell culture medium. The results indicated that RAW264.7 cells treated with MQZP exhibited a notable decrease in the secretion of IL-1β, TNF-α, and MCP-1 when contrasted with the ox-LDL model group (Fig. **7A**-**C**), suggesting that MQZP may alleviate the inflammatory response linked to foam cell formation.

To assess whether the protective effects of MQZP against AS are connected to the key targets and pathways predicted by network pharmacology, we examined the mRNA expression of TP53, SRC, STAT3, and IL-6, along with the protein expression of IL-6, STAT3, and p-STAT3 in RAW264.7 cells. The ox-LDL group demonstrated a marked increase in mRNA expression of TP53, SRC, STAT3, and IL-6 compared to the control group, but this elevation was significantly reduced after treatment with MQZP (Fig. **7D**-**G**). STAT3, a key target highlighted in the network pharmacology analysis, is pivotal in the IL-6/STAT3 signaling pathway, which plays a crucial role in the regulation of inflammation. The ox-LDL group displayed significant upregulation of IL-6, STAT3, and p-STAT3 proteins, while their expression levels were considerably lowered following MQZP treatment (Fig. **7H**, **I**). The findings imply that MQZP may help combat AS by blocking the IL-6/STAT3 pathway activation and lowering inflammation.

## 4 DISCUSSION

TCM formulations differ from individual active components found in conventional chemical drugs. Moreover, each TCM formulation consists of multiple ingredients, forming a complex system composed of numerous compounds [39]. Furthermore, the benefits of TCM formulations lie in their multi-link, multi-target mechanism of action, potentially complicating TCM research processes, a dilemma that seems to have been alleviated by the recent emergence of network pharmacology. With its systematic perspective and advantages in multi-level interactions, network pharmacology offers a novel avenue for studying complex TCM formulations [40]. For instance, through network pharmacology, the Yangyinghuoxue, Qingxin Lianzi Yin, and Daqinjiao decoctions were found to alleviate liver fibrosis, diabetic nephropathy, and cerebral small vessel diseases, respectively [41-43]. The pathological mechanisms of AS involve complex processes, including chronic arterial intima damage, inflammatory reactions, foam cell and atheromatous plaque formation, plaque instability and rupture, thrombosis formation, and arterial narrowing and blockage [3]. These pathological processes are intertwined, making AS pathogenesis complex and multi-layered. These characteristics have also led to an increasing preference for multi-component, multi-target TCM formulations for AS treatment. Moreover, a growing body of research is exploring the mechanisms of these formulations through network pharmacological analysis.

Currently, traditional Chinese medicines with anti-inflammatory effects are widely used in clinical practice to treat various inflammation-related diseases. Studies have confirmed that snow lotus can reduce inflammation and joint damage in arthritis rats [20]. Research by Gong *et al*. also found that snow lotus alleviates chronic inflammation through the NF-κB/PI3K/MAPK signaling pathway [44]. Scutellaria baicalensis regulates immune responses and reduces inflammation by inhibiting the production of inflammatory factors such as TNF-α, IL-1, IL-6, and COX-2, and is widely used in the treatment of inflammatory bowel disease, rheumatoid arthritis, hepatitis, and other conditions [45]. Glycyrrhiza uralensis, one of the most commonly used herbs in traditional Chinese medicine, has significant anti-inflammatory and immune-regulatory effects. It exerts its anti-inflammatory action by reducing TNF-α and IL-6 secretion, inhibiting the NF-κB pathway, and regulating immune function, thereby improving osteoarthritis [46]. Additionally, studies indicate that licorice extract can target the TGF-β1/Smad/Stat3 pathway to alleviate high glucose-induced renal tubular fibrosis [47]. Furthermore, with the advancement of modern research in traditional Chinese medicine, an increasing number of herbs with cardiovascular protective effects have been discovered and applied in the prevention and treatment of cardiovascular diseases. For example, Dioscorea not only enhances anticoagulant function, inhibits platelet aggregation and thrombosis, but also reduces oxidative stress and apoptosis in cardiomyocytes, thus delaying the progression of cardiovascular diseases [48, 49]. Salvia miltiorrhiza is widely used in the treatment of cardiovascular diseases for its effects on activating blood circulation, dissipating stasis, relieving pain, and improving microcirculation [18, 19]. Zhao *et al*. combined Astragalus membranaceus-Carthamus tinctorius and found that this combination significantly improved cerebral ischemia-reperfusion injury [50].

Qianzheng Powder is a well-known TCM formulation. Some clinical studies have shown that many of the herbs in the MQZP formula exert their effects through multiple bioactive components, targets, pathways, and mechanisms [51]. These include alleviating inflammation, promoting cell repair, dilating blood vessels, improving microcirculation, and relieving smooth muscle spasms [52, 53]. For example, Qianzheng Powder has been shown to improve serum levels of Lp-PLA2, NF-κB, IL-2, IL-6, and TNF-α in patients with acute ischemic stroke, inhibiting inflammation and improving hemodynamics [54]. The success of these applications motivated us to investigate the possible mechanism of MQZP in the treatment of AS using network pharmacological analysis. Our findings revealed 124 active MQZP components acting on 417 disease-related targets associated with AS. Drawing from the topological analysis of the "herb-ingredient-target" network, cyclo(D)-Pro-(D)-Phe, aurantiamide, beauverilide a, ecdysone, cyclo (D)-Pro-(D)-Ile, coumarins, cyclo(D)-Pro-(D)-Val, Byakangelicol, palmitic acid, and chitinase were highly correlated with relevant targets, potentially serving as the key active components in MQZP. Notably, Cyclo(D)-Pro-(D)-Phe, cyclo (D)-Pro-(D)-Ile, and cyclo(D)-Pro-(D)-Val belong to the dd-diketopiperazines class of compounds, and previous research demonstrated their anti-tumor, antiviral, and antibacterial properties [55], among other mechanisms associated with anti-hyperglycemia [56], anti-aging [57], and neuroprotection effects [58]. Besides alleviating inflammatory reactions through the inhibition of the NF-κB signaling pathway and p38 MAPK phosphorylation [59], Aurantiamide could also suppress PTGS2 expression [60]. On the other hand, Beauverilide a demonstrated remarkable efficacy in combating AS, with the specific mechanisms involving reduction of lipid accumulation in macrophages [61], ACAT-1 and ACAT-2 inhibition, and alleviation of atherosclerotic plaques in the hearts and aortas of ApoE-/- mice [62].

The core targets identified from the PPI network included TP53, SRC, STAT3, and AKT1, which are linked to the initiation and development of AS. TP53, a tumor suppressor gene, regulates vascular endothelial cell homeostasis and inflammation [63]. However, under pathologically overactive conditions, TP53's excessive activation exacerbates AS progression [64]. In addition, the SRC kinase regulates cell signaling pathways during AS progression, potentially accelerating plaque formation and impacting the integrity and function of the vascular wall [65]. On the other hand, STAT3 is a part of the cellular signaling pathway, and abnormal STAT3 activation could contribute to AS development *via* pathways such as endothelial dysfunction, macrophage polarization, and inflammation [30]. Finally, AKT1 serves as an essential part of the PI3K/AKT signaling pathway while modulating AS pathogenesis by regulating various BPs, including cell survival, proliferation, differentiation, and metabolism [66].

Based on the findings mentioned above, we conclude that the essential effective components and core targets within the “herb-ingredient-target” network can help prevent and treat AS. Consequently, we performed GO enrichment analysis along with KEGG pathway enrichment analysis. The results of the GO enrichment analysis indicated that the 417 potential therapeutic targets were primarily concentrated in BP. During the KEGG pathway enrichment analysis, 238 pathways were found to be enriched with the 417 possible therapeutic targets. Among them, 195 targets were enriched in the lipid and atherosclerosis pathway, highlighting the pivotal role of lipid metabolism in the development of AS. Lipid regulation remains a major therapeutic strategy in clinical practice. These research findings are consistent with our previous speculation, demonstrating the crucial role of lipid metabolism in the anti-atherosclerotic mechanism of MQZP.

Moreover, our study suggests that targeting the IL-6/STAT3 pathway may be a promising therapeutic approach for MQZP in the treatment of AS. The IL-6/STAT3 pathway plays a critical role in AS, involving multiple processes such as inflammation, smooth muscle cell proliferation, lipid metabolism dysregulation, endothelial dysfunction, and thrombosis. Activation of STAT3 upregulates the expression of GSDME, thereby exacerbating the inflammatory response in AS through increased pyroptosis [67]. Additionally, reducing METTL3 levels regulates the JAK2/STAT3 pathway through modulation of IGF2BP1, alleviating lipid accumulation in AS [68]. miR-15a-5p-CX3CL1 and STAT3 promote AS progression by enhancing endothelial cell proliferation, migration, and inflammation [69]. Raloxifene inhibits vascular smooth muscle proliferation and mitigates high-fat diet-induced AS in ApoE−/− mice by suppressing the IL-6/STAT3 signaling pathway [29]. Akita *et al*. also mentioned that inhibiting the IL-6/STAT3 signaling pathway alleviates AS caused by dyslipidemia and inflammation [70]. Furthermore, studies have shown that traditional Chinese medicine or its extracts can regulate AS through the STAT3 pathway. For example, it has been found that Xie Ling Dan relieves AS by inhibiting STAT3 phosphorylation and the MAPK signaling pathway [33]. Buyang Huanwu Decoction downregulates the JAK/STAT3 signaling pathway to reduce AS-related inflammation and lipid deposition [71]. An enzyme extracted from barley bran reduces cellular lipid uptake and the secretion of inflammatory factors by downregulating CD36 and STAT3 expression [72].

Previous findings were derived from network pharmacology analysis. Thus, we aimed to validate and confirm these findings through experimental methods. To achieve this, we utilized computational biology techniques, particularly molecular docking, and carried out *in vitro* experiments to validate the predictions generated by network pharmacology. Molecular docking results showed stable binding of TP53, SRC, STAT3, AKT1, JUN, and IL-6 with their corresponding compounds. This stable binding suggests that these compounds may be potential drug candidates, which can interact with specific protein targets, thereby influencing biological processes. *In vitro* experimental results indicated that MQZP suppressed the levels of TP53, SRC, STAT3, and IL-6 mRNA, inhibited the IL-6/STAT3 signaling pathway, and alleviated the inflammatory response during the progression of AS.

## 5 STUDY LIMITATIONS

However, this study is not without limitations. The limitations of this study primarily lie in two aspects: First, we utilized public databases, such as TCMSP, GeneCards, and DisGeNET. The update frequency and timeliness of these databases may lag behind the latest research, and the focus of each database may differ, which could pose potential risks. Second, although the aforementioned research suggests that MQZP has a certain therapeutic effect on AS, this conclusion is primarily based on network pharmacology data analysis and *in vitro* experimental validation. This is not comprehensive enough, and further *in vitro* studies and clinical research are needed to validate the findings.

## CONCLUSION

In this experimental study, we verified that MQZP can play an anti-atherosclerotic role by reducing the macrophage inflammatory response. The key mechanism may be related to the reduction of lipid accumulation in macrophages and the inhibition of the IL-6/STAT3 signaling pathway. This study clarified the potential pharmacological mechanism of MQZP in improving AS, and provided a theoretical basis and experimental data for the clinical application of MQZP in AS.

## Figures and Tables

**Fig. (5):**
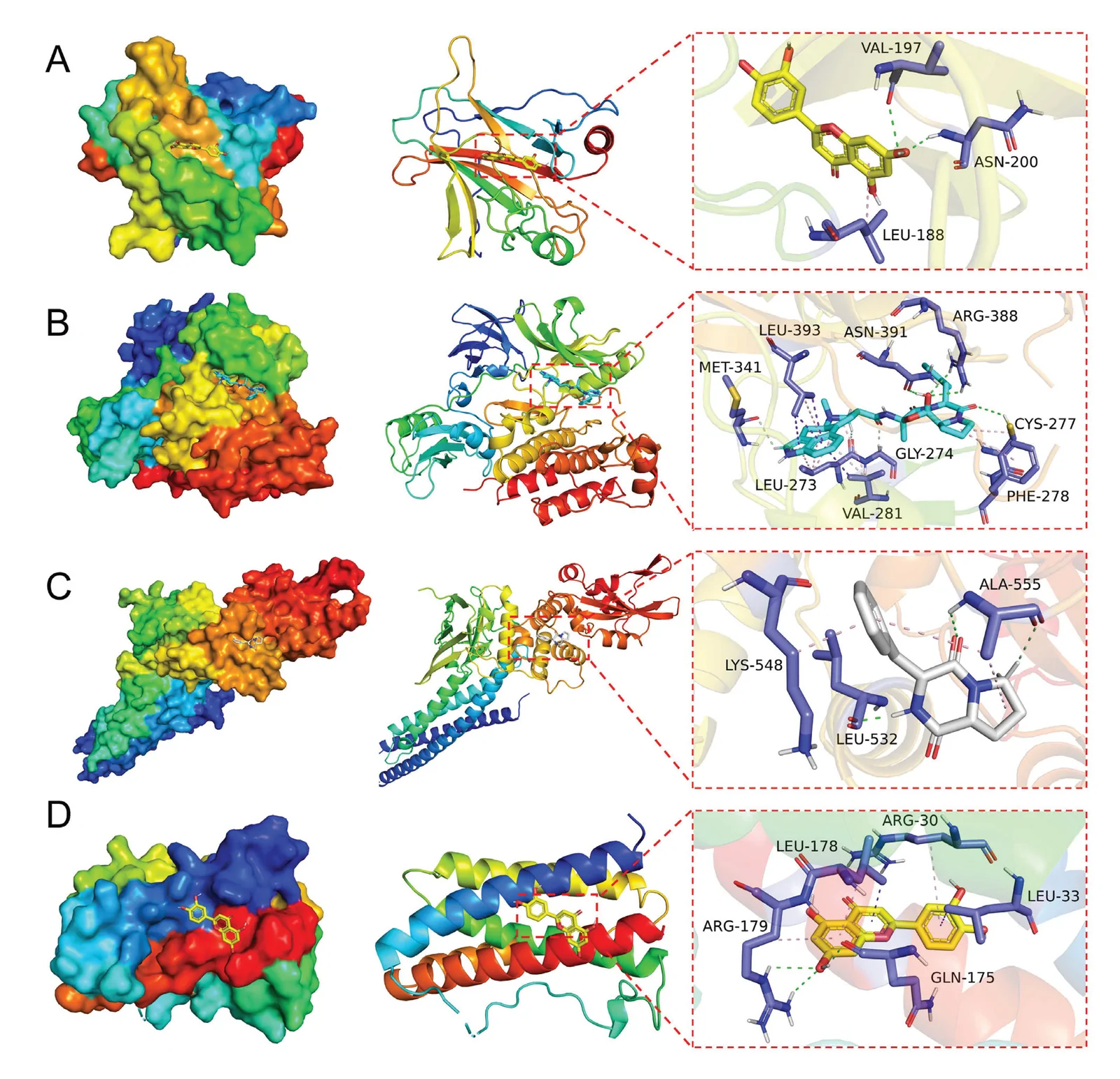
Visualization of molecular docking results. (**A**) A docking diagram of TP53-Luteolin. Luteolin establishes two conventional hydrogen bonds with the amino acid residues ASN-200 and VAL-197 in TP53 and a π-Alkyl interaction with LEU-188. (**B**) The docking diagram of SRC-ergotamine. Ergotamine establishes three conventional hydrogen bonds with the amino acid residues CYS-277, ARG-388, and ASN-391 in SRC, three carbon-hydrogen bonds with GLY-274, LEU-273, and MET-341, and eight Alkyl interactions with PHE-278, CYS-277, VAL-281, LEU-393, and LEU-273. It also forms two π-σ interactions with VAL-281 and LEU-393. (**C**) The docking diagram of STAT3-cyclo-D--Pro-D-Phe. Cyclo-D--Pro-D-Phe establishes two conventional hydrogen bonds with the amino acid residues ALA-555 and LEU-532 in STAT3, a carbon-hydrogen bond with ALA-555, and three Alkyl interactions with ALA-555 and LYS-548. (**D**) The docking diagram of IL-6-Luteolin. Luteolin establishes two conventional hydrogen bonds with the amino acid residues GLN-175 and ARG-179 in IL-6 and three π-Alkyl interactions with ARG-30, ARG-179, and LEU-178. It also creates two π-σ interactions with LEU-33 and LEU-178. 3.6 MQZP promotes RAW264.7 cell viability and ameliorates ox-LDL-induced lipid accumulation.

**Table 1:** Primer sequences for RT-qPCR.

**Genes**	**Sequences (5’-3’)**
TP53	TGGAAGGAAATTTGTATCCCGA (forward)
	GTGGATGGTGGTATACTCAGAG (reverse)
SRC	CTATGTGGAGCGGATGAACTAT (forward)
	ATTCGTTGTCTTCTATGAGCCG (reverse)
STAT3	TGTCAGATCACATGGGCTAAAT (forward)
	GGTCGATGATATTGTCTAGCCA (reverse)
IL-6	CTCCCAACAGACCTGTCTATAC (forward)
	CCATTGCACAACTCTTTTCTCA (reverse)

**Table 2:** Details of MQZF compound Chinese medicine.

**Chinese Name**	**English Name**	**Latin Name**	**Medicament Portions**	**Ratio in MQZF**
Baifuzi	Giant Typhonium Tuber	Typhonii Rhizoma	Root, rhizome	10%
Baijiangcan	Stiff Silkworm	Bombyx Batryticatus	Insect body	15%
Quanxie	Scorpion	Scorpio	Insect body	5%
Chishao	Red Paeoniae Trichocarpae	Paeoniae Radix Rubra	Root, rhizome	30%
Danggui	Chinese Angelica	Angelicae Sinensis Radix	Root, rhizome	15%
Chuanxiong	Szechwan Lovage Rhizome	Chuanxiong Rhizoma	Root, rhizome	15%
Jixueteng	Suberect Spatholobus Stem	Spatholobi Caulis	Stem	10%

**Table 3:** The topological parameters of the key effective ingredients.

**No.**	**Ingredient Name**	**Molecular Function**	**Degree**	**BC**	**CC**
1.	cyclo(D)-Pro-(D)-Phe	C14H16N2O2	60	15389.93	0.37
2.	aurantiamide	C25H26N2O3	57	13134.94	0.38
3.	beauverilide a	C31H49N3O5	56	16628.63	0.37
4.	ecdysone	C27H44O6	52	12044.48	0.38
5.	cyclo(D)-Pro-(D)-Ile	C11H18N2O2	52	9576.05	0.37
6.	cyclo(D)-Pro-(D)-Val	C10H16N2O2	51	8658.7	0.37
7.	coumarins	C19H16O4	51	11217.65	0.37
8.	palmitic acid	C16H32O2	48	7324.92	0.37
9.	chitinase	C20H21N5O2S	48	9562.64	0.37
10.	Byakangelicol	C17H16O6	48	8859.10	0.37

## Data Availability

The datasets used and/or analysed during the current study were publicly available from TCMSP Database (https://tcmsp.91medicine.cn/#/home), HERB database (http://herb.ac.cn/), SwissTargetPrediction database (http://swisstargetprediction.ch/), GeneCards (https://www.genecards.org/), DisGeNET (https://www.disgenet.org/), OMIM (https://omim.org/), DrugBank (https://go.drugbank.com/), STRING database (https://cn.string-db.org/), Metascape database (http://metascape.org/), UniProt (https://www.uniprot.org/), PDB (http://www1.rcsb.org/), PubChem (https://pubchem.ncbi.nlm.nih.gov/).

## LIST OF ABBREVIATIONS

AKT1: AKT Serine/Threonine Kinase 1
AS: Atherosclerosis
BC: Betweenness Centrality
BCA: Bicinchoninic Acid
BP: Biological Process
CC: Closeness Centrality
CCK-8: Cell Counting Kit-8
COX-2: Cyclooxygenase-2
CTNNB1: Catenin Beta 1
DC: Degree Centrality
DMEM: Dulbecco's Modified Eagle Medium
ELISA: Enzyme-Linked Immunosorbent Assay
FBS: Fetal Bovine Serum
GO: Gene Ontology
IGF2BP1: Insulin Like Growth Factor 2 MRNA Binding Protein 1
IL-1β: Interleukin-1 Beta
IL-6: Interleukin-6
JUN: Jun Proto-Oncogene
KEGG: Kyoto Encyclopedia of Genes and Genomes
Lp-PLA2: Lipoprotein-Associated Phospholipase A2
MAPK1: Mitogen-Activated Protein Kinase 1
MAPK3: Mitogen-Activated Protein Kinase 3
MCP-1: Monocyte Chemoattractant Protein-1
METTL3: Methyltransferase Like 3
MNC: Maximal Clique Centrality
MQZP: Modified Qianzheng Powder
NF-κB: Nuclear Factor Kappa B
OMIM: Online Mendelian Inheritance in Man
ox-LDL: Oxidized Low-Density Lipoprotein
PI3K: Phosphatidylinositol-3-Kinase
PIK3CA: Phosphatidylinositol-4,5-Bisphosphate 3-Kinase Catalytic Subunit Alpha
PIK3CB: Phosphatidylinositol-4,5-Bisphosphate 3-Kinase Catalytic Subunit Beta
PPI: Protein-Protein Interaction
PTGS2: Prostaglandin-Endoperoxide Synthase 2
PVDF: Polyvinylidene Difluoride
RIPA: Radioimmunoprecipitation Assay
RT-qPCR: Reverse Transcription Quantitative Polymerase Chain Reaction
SDS-PAGE: Sodium Dodecyl Sulfate Polyacrylamide Gel Electrophoresis
SRC: SRC Proto-Oncogene
STAT3: Signal Transducer and Activator of Transcription 3
TCMSP: Traditional Chinese Medicine Systems Pharmacology Database and Analysis Platform
TNF-α: Tumor Necrosis Factor Alpha
TP53: Tumor Protein p53
