# Supplementary material for: Network Pharmacology and Experimental Validation of the Anti-Inflammatory Effect of Modified Qianzheng Powder in Atherosclerosis
Source: Comb Chem High Throughput Screen. 2025 Jul 3;29(7):1181–98. doi: 10.2174/0113862073376112250602092950 (PMC13539591; doi:10.2174/0113862073376112250602092950)
Supplement: Supplementary file 1 [file CCHTS-29-7-1181_SD1.pdf]

Supplementary Material

Network Pharmacology and Experimental Validation of the Anti-Inflammatory Effect of Modified Qianzheng Powder in Atherosclerosis

Nianpei Yin[1,2,#], Hui Luo[3,#], Jie Feng[2], Weisheng Zhan[1], Zheng Zhou[1] and Ying Yang[2,*]

[1]Department of Clinical Medicine, North Sichuan Medical College, Nanchong 637000, Sichuan, China; [2]Department of Cardiovascular Disease, Affiliated Hospital of North Sichuan Medical College, Nanchong 637000, Sichuan, China; [3]Department of Thoracic Surgery, Second Clinical College of Nanchong North Sichuan Medical College, Nanchong Central Hospital, Nanchong 637000, Sichuan, China

Table S1. Details of 124 active compounds of MQZF.

ID	Compound	Herb	MW	OB	DL
MOL000359	sitosterol	BFZ、CX<br>BFZ、CX<br>BFZ、CS、DG、JXT<br>BFZ<br>BFZ<br>BFZ<br>BFZ<br>BFZ、DG<br>BFZ<br>BFZ<br>BFZ、BJC、QX<br>BFZ<br>BJC<br>BJC<br>BJC<br>BJC<br>BJC<br>BJC<br>BJC<br>BJC<br>BJC<br>BJC<br>BJC<br>BJC<br>BJC<br>BJC<br>BJC<br>BJC<br>BJC	414.79	36.91	0.75
MOL001494	Mandenol		308.56	42	0.19
MOL000358	beta-sitosterol		414.79	36.91	0.75
MOL001788	adenine		308.56	62.81	0.03
MOL001454	berberine		336.39	36.86	0.78
/	campesteryl glucoside		/	/	/
MOL001458	coptisine		320.34	30.67	0.86
MOL000676	dibutyl phthalate		278.38	64.54	0.13
MOL000041	L-phenylalanine		165.21	41.62	0.04
MOL000421	nicotinic acid		123.12	47.65	0.02
MOL000069	palmitic acid		256.48	19.3	0.1
MOL010716	xanthine		152.13	46.17	0.04
HBIN017353	aurantiamide		444.57	58.02	0.52
HBIN017672	beauverilide a		/	/	/
HBIN019100	butyl-2-pyrrolidone-5-carboxylate		/	/	/
HBIN020341	chitinase		/	/	/
HBIN022204	cyclo(D)-Pro-(D)-Phe		/	/	/
HBIN022205	cyclo(D)-Pro-(D)-Val		/	/	/
HBIN024784	ecdysone		480.71	6.94	0.82
HBIN024786	ecdysterone		480.71	5.3	0.82
HBIN025558	ergotamine		581.73	8.1	0.21
HBIN030901	isololiolide		/	/	/
HBIN038678	palmitamide		255.50	19.79	0.1
HBIN040007	(+)-pinoresinol		358.42	4.25	0.52

HBIN044728	Stearamide	QX	283.56	18.44	0.14
HBIN045891	tenellin	QX<br>QX	/	/	/
HBIN022203	cyclo (D)-Pro-(D)-Ile	QX	/	/	/
/	(-)-medioresinol	CS	/	/	/
/	(-)-lariciresinol	CS	/	/	/
HBIN044730	stearic acid	CS<br>CS	284.54	17.83	0.14
MOL011455	20-hexadecanoylingenol	CS	418.58	32.7	0.65
MOL000953	cholesterol	CS、DG、JXT	386.73	37.87	0.68
MOL002156	trimethylamine	CS、JXT	59.13	59.98	0
MOL001002	ellagic acid	CS<br>CS	302.20	43.06	0.43
MOL001924	paeoniflorin	CS	480.51	53.87	0.79
MOL002714	baicalein	CS	270.25	33.52	0.21
MOL002776	Baicalin	CS<br>CS	446.39	40.12	0.75
MOL004355	Spinasterol	CS	412.77	42.98	0.76
MOL000449	Stigmasterol	CS	412.77	43.83	0.76
MOL000492	(+)-catechin	CS	290.29	54.83	0.24
MOL006992	(2R,3R)-4-methoxyl-distylin	CS<br>CS	318.30	59.98	0.3
MOL001921	Lactiflorin	CS	462.49	49.12	0.8
MOL001925	paeoniflorin_qt	CS	318.35	68.18	0.4
MOL007003	benzoyl paeoniflorin	CS	584.62	31.14	0.54
MOL007004	Albiflorin	CS<br>CS	480.51	30.25	0.77
MOL007008	4-ethyl-paeoniflorin_qt	CS	332.38	56.87	0.44
MOL007012	4-o-methyl-paeoniflorin_qt	DG	332.38	56.7	0.43
MOL007014	8-debenzoylpaeonidanin	DG<br>DG	390.43	31.74	0.45
MOL007016	Paeoniflorigenone	DG	318.35	65.33	0.37
MOL007025	isobenzoylpaeoniflorin	DG	584.62	31.14	0.54
/	N-Propyl Gallate	DG	/	/	/
MOL000103	benzoic acid	DG<br>DG	138.13	30.15	0.03
/	Palbinone	DG	/	/	/
/	Propylparaben	DG	/	/	/
/	Queen Bee acid	DG	/	/	/
MOL000098	Quercetin	DG<br>DG	302.25	46.43	0.28
MOL003138	Swertianin	DG	274.24	28.67	0.24
/	1,2-Benzenedicarboxylic acid	DG	/	/	/
MOL003507	1-Heptanol	DG<br>DG	116.23	17.22	0.01
/	2-Butenoicacid,2-methyl-, (Z)-	DG	/	/	/
/	2-Hydroxy-5-methyl acetophenone	DG	/	/	/
MOL003590	Angelicin	DG、CX	186.17	19.6	0.1

/	Angenomalin	DG	/	/	/
MOL001945	bergapten	DG	216.20	42.21	0.13
MOL000244	Borneol	CX	154.28	81.8	0.05
MOL005800	Byakangelicol	CX	316.33	41.42	0.36
MOL001956	cnidilin	CX	300.33	32.69	0.28
MOL002905	Columbianadin	CX	328.39	14.82	0.36
MOL000431	coumarin	CX	146.15	29.17	0.04
MOL013077	Decursin	CX	328.39	39.27	0.38
MOL000666	Hexanal	CX	100.18	55.71	0.01
MOL001941	imperatorin	CX	270.30	34.55	0.22
/	Isobornyl acetate	CX	/	/	/
MOL001942	isoimperatorin	CX	270.30	45.46	0.23
/	Isooxypeucedanin	CX	/	/	/
MOL003561	Isopimpinellin	CX	246.23	25.93	0.17
MOL011782	Ligustilide	CX	190.26	23.5	0.07
/	Meranzin	CX	/	/	/
MOL000879	Methyl hexadecanoate	JXT	270.51	18.09	0.12
MOL002135	Myricanone	JXT	356.45	40.6	0.51
MOL002140	Perlolyrine	JXT	264.30	65.95	0.27
MOL002157	wallichilide	JXT	412.57	42.31	0.71
MOL000433	FA	JXT	441.45	68.96	0.71
MOL002151	senkyunone	JXT	326.52	47.66	0.24
MOL000389	cis-Ferulic acid	JXT	194.20	54.97	0.06
/	lignans	JXT	/	/	/
MOL000635	Vanillin	JXT	152.16	52	0.03
MOL000414	Caffeic acid	JXT	180.17	54.97	0.05
MOL008288	Coniferyl ferulate	JXT	356.40	4.54	0.39
MOL000431	coumarins	JXT	146.15	29.17	0.04
MOL004265	Ethyl ferulate	JXT	222.26	10.24	0.08
MOL000360	ferulic acid	JXT	194.20	39.56	0.06
MOL002208	Senkyunolide A	JXT	192.28	26.56	0.07
MOL000296	hederagenin	JXT	414.79	36.91	0.75
MOL000392	formononetin	JXT	268.28	69.67	0.21
MOL000417	Calycosin	JXT	284.28	47.75	0.24
MOL000461	3,7-dihydroxy-6-methoxy-dihydroflavonol	JXT	302.30	43.8	0.26
MOL000468	8-o-Methylreyusi		298.31	70.32	0.27
MOL000470	8-C-α-L-arabinosylluteolin		418.38	35.54	0.66
MOL000471	aloe-emodin		270.25	83.38	0.24

MOL000483	(Z)-3-(4-hydroxy-3-methoxy-phenyl)-N-[2-(4-hydroxyphenyl) ethyl] acrylamide		313.38	118.35	0.26
MOL000490	petunidin		317.29	30.05	0.31
MOL000493	campesterol		400.76	37.58	0.71
MOL000497	licochalcone a		338.43	40.79	0.29
MOL000500	Vestitol		272.32	74.66	0.21
MOL000501	Consume close grain		302.30	68.12	0.27
MOL000502	Cajinin		300.28	68.8	0.27
MOL000503	Medicagol		296.24	57.49	0.6
MOL000507	Psi-Baptigenin		282.26	70.12	0.31
MOL000006	luteolin		286.25	36.16	0.25
MOL000491	Augelicin		426.50	37.5	0.66
MOL000390	Daidzein		254.25	19.44	0.19

Table S2. Information of 9 core targets.

Gene Symbol	Protein Name	Degree	BC	CC
TP53	Cellular tumor antigen p53	54	14064.79	0.27
SRC	Proto-oncogene tyrosine-protein kinase Src	43	9938.1	0.30
STAT3	Signal transducer and activator of transcription 3	41	7140.46	0.33
AKT1	RAC-alpha serine/threonine-protein kinase	37	4213.38	0.31
JUN	Transcription factor Jun	32	4999.78	0.36
PIK3CA	Phosphatidylinositol 4,5-bisphosphate 3-kinase catalytic subunit alpha isoform	32	2906.48	0.32
MAPK1	Mitogen-activated protein kinase 1	31	3290.75	0.31
CTNNB1	Catenin beta-1	31	5048.41	0.32
MAPK3	Mitogen-activated protein kinase 3	30	3290.47	0.34

Table S3. Molecular docking energy of core targets and corresponding compounds.

Target / Compound	Binding Energy (kcal/mol)					
	**TP53**	**SRC**	**STAT3**	**AKT1**	**JUN**	**IL-6**
Aloe-emodin	-6.2	/	/	/	/	/
baicalein	-5.9	/	/	-9.8	/	/
Luteolin	-6.3	/	/	-10.3	-6.1	-7.2
Albiflorin	/	-8.2	/	/	/	/
Angenomalin	/	-8.1	/	/	/	/
Aurantiamide	/	-8.3	/	/	/	/
Beauverilide-A	/	-7.6	/	/	/	/
Berberine	/	-8.7	/	/	/	/
butyl-2-pyrrolidone-5-carboxylate	/	-5.3	/	/	/	/
Chitinase-IN-2	/	-8.8	/	-10.4	/	/

Columbianadin	/	-8.1	/	/	/	/
Coniferyl-ferulate	/	-7.8	-6.1	/	/	/
coumarins	/	-7.1	/	-9.3	/	/
Decursin	/	-8.6	/	/	/	/
ergotamine	/	-9.7	/	/	/	/
N-Propyl-Gallate	/	-5.9	/	/	/	/
quercetin	/	-8.7	/	-10.4	/	/
Caffeic-acid	/	/	-5.5	/	/	/
campesteryl-glucoside	/	/	-6.3	/	/	/
cis-Ferulic-acid	/	/	-5.3	/	/	/
cyclo-D--Pro-D-Phe	/	/	-6.5	-8.5	/	/
Ethyl-ferulate	/	/	-5.3	/	/	/
ferulic-acid	/	/	-5.4	/	/	/
Licochalcone-A	/	/	-6.3	/	/	/
senkyunone	/	/	-5.6	/	/	/
2-Butenoicacid	/	/	/	-4.5	/	/
Isopimpinellin	/	/	/	-8.2	/	/
Propylparaben	/	/	/	-6.5	/	/
formononetin	/	/	/	/	-6.4	/
Paeoniflorigenone	/	/	/	/	-7	/
ecdysone	/	/	/	/	/	-7.1
Paeoniflorin	/	/	/	/	/	-6.6
